# Lived experiences of transgender forced migrants and their mental health outcomes: systematic review and meta-ethnography

**DOI:** 10.1192/bjo.2022.51

**Published:** 2022-05-10

**Authors:** Susannah Hermaszewska, Angela Sweeney, B Camminga, Riley Botelle, Kate Elliott, Jacqueline Sin

**Affiliations:** School of Health Sciences, City University of London, UK; Department of Health Service and Population Research, King's College London, UK; African Centre for Migration & Society, University of the Witwatersrand, Johannesburg, South Africa; Royal Berkshire Hospital, Reading, UK; East London NHS Foundation Trust, UK; School of Health Sciences, City University of London, UK

**Keywords:** Qualitative research, refugee, migration, transgender, mental health

## Abstract

**Background:**

Owing to multiple, complex and intersecting health inequities, systemic oppression and violence and discrimination in their home countries, some transgender people are forced to migrate to countries that offer them better legal protection and wider social acceptance.

**Aims:**

This review sought to explore and understand the multiple factors that shape the mental health outcomes of transgender forced migrants (TFMs).

**Method:**

We systematically searched nine electronic databases for multidisciplinary literature (PROSPERO ID: CRD42020183062). We used a meta-ethnographic approach to synthesise data. We completed a quality appraisal and developed a socio-ecological model to draw together our findings.

**Results:**

We retrieved 3399 records and screened titles, abstracts and full text to include 24 qualitative studies in this review. The synthesis identified individual survival strategies and factors in interpersonal, organisational and societal environments that contributed to profound deprivation and mental distress in TFMs. Pervasive and persistent violence and discrimination, economic exclusion, barriers to healthcare and a dependency on legal documentation were identified as key factors leading to poor mental health outcomes. Sources of resilience included community acceptance and support, being granted asylum, societal affirmation of gender, fulfilment of basic rights and healthcare access. Individual strategies for survival, such as hope and having purpose in life, were important in bringing relief from distress.

**Conclusions:**

Improved communication and knowledge about the unique needs and concerns of TFMs through interventions at the individual, interpersonal, organisational and societal levels are necessary to improve mental health outcomes.

## Health disparities of transgender people

Transgender people, who are estimated to make up 0.1–2.0% of the population and around 8% of the lesbian, gay, bisexual and transgender (LGBT) population,^1^ have a gender identity or expression that differs from the sex they were assigned at birth. Globally, transgender people face multiple, complex and intersecting health inequities including systematic social and economic marginalisation, pathologisation, stigma, discrimination, and violence across their lifespan.^2,3^ Depending on the country they reside in, transgender people experience varying levels of systematic oppression and lack of statutory protection, which further exposes them to violence and perpetuates social exclusion.^4^ These stressors are linked to poor mental health outcomes, including disproportionate rates of suicide attempts (twice those of cisgender lesbian, gay and bisexual adults and ten times those of the general adult population) and diagnosed mental health conditions such as depression, anxiety and substance use disorders.^5–9^ In the worst cases, transgender people are forced to migrate to seek refuge in countries that offer them better legal protection, perceived safety and access to healthcare.

## Transgender forced migration

Intersectionality is the term used to describe how multiple marginalised social identities operate together and exacerbate inequality.^10^ Forced migration increases risk factors for poor mental health outcomes, including lack of access to basic necessities for survival, lack of certainty about the future, detention, fear of asylum claim rejection, and barriers to accessing healthcare during the journey and upon arrival at a host country.^11–13^ Health research on transgender forced migrants (TFMs) primarily situates this population within the broader umbrella of LGBT and Queer (LGBTQ+) forced migrants. Recent qualitative work has revealed the complex role of additional stressors experienced by this population in relation to their LGBTQ+ identities, such as complex childhood abuse, lifelong victimisation, abuse by immigration officials and other state actors, and systematic barriers during the asylum-seeking process.^14–22^ These additional stressors may explain the higher incidence of mental distress in LGBTQ+ forced migrants relative to the overall forced migrant population.^23^ The intersectional experience of being a TFM must be taken into consideration when researching and working with this population.

## Resilience and resourcefulness of TFMs

Qualitative work has also highlighted coping strategies used by LGBTQ+ migrants, including staying positive and fostering migration aspirations; utilising community and legal support; peer and partner support; concealment strategies; resignation to life circumstances; and avoidance behaviours.^16,24,25^ This has contributed to a shifting narrative in health research that frames forced migrants not just in terms of their ‘victimhood’ but also in terms of their agency, resourcefulness and resilience.^26,27^ This has important implications for clinical research and practice as it suggests that ongoing distress and post-traumatic growth can coexist.^28^ These coping strategies can inform the design of evidence-based interventions.^29^

## Limitations of existing research body

However, a key limitation of relying on the umbrella group of LGBTQ+ in health research is that it elides the specificity of needs and experiences of individual population groups.^30^ Studies on LGBTQ+ people typically have fewer than 25% transgender participants.^31^ As a result, findings often underemphasise or omit lived experiences that are unique to transgender people, such as transphobia, gender dysphoria, and barriers to accessing gender inclusive and affirming care. Considering the impact of being transgender can contribute to a richer understanding of the factors affecting mental health outcomes in this population; leaving such considerations out can expose individuals to further risks and vulnerabilities and perpetuate or exacerbate inequalities. To date, there has been no evidence synthesis on the lived experiences of TFMs.

## Aims and focus of the meta-ethnography

This review aimed to draw together multidisciplinary literature exploring the lived experiences of TFMs using a meta-ethnographic approach.^32^ We sought to explore and understand the multiple factors that shape their mental health outcomes, including high rates of suicide, mental health diagnoses and distress, that arise when transgender people are forced to migrate across national borders, as well as the barriers and facilitators transgender people face when accessing healthcare during migration and in their host countries. Following study selection, it was apparent that the data were sufficiently rich to inform the development of a theoretical model; this therefore formed a further aim, with the hope that this will shape future research, policy and health interventions in this field.

## Method

Meta-ethnography has the potential to produce new conceptual insights about complex phenomenon while also exploring research contexts and processes.^32^ This method can therefore be used to form a coherent global narrative of an emerging field that is critical of the role of knowledge-producing processes and the social and political contexts in which the research is produced. Meta-ethnography emphasises the preservation of the context and meaning of the primary studies.^33^ This is enabled by the distinction of data into first-order (participant quotations), second-order (author interpretations) and third-order (reviewer interpretations) data.^34^ This synthesis method allowed us to separately consider the lived experiences of TFMs foremost and also the second-order interpretations of such data by researchers.

The review protocol was prospectively registered on PROSPERO.^35^ The review process followed the PRISMA guidelines^36^ and reporting adheres to the eMERGe guidance for reporting meta-ethnography.^37^

### Positionality

The review team comprised of a partnership of researchers from different disciplines, backgrounds and standpoints. S.H. is a White, cisgender woman with a history of ancestral forced migration and trauma. She conducts research in partnership with refugees to understand and overcome barriers to mental healthcare and recovery from trauma. A.S. is a White, cisgender trauma survivor researcher who uses participatory methods in a survivor research framework to prioritise and explore experiential knowledge. B.C. is a White transgender South African who works predominantly in the field of migration and refugee studies with a specific focus on transgender migrants from the African continent. R.B. is a non-migrant, White queer transmasculine person working as an academic and medical doctor in England. He actively works on being critical about biomedical approaches and prioritising centring voices of people as experts in their own experiences. K.E. is a cisgender, mental health social worker in London. She has a background in sociological research promoting patient involvement in decision-making around their healthcare. J.S. is a cisgender, academic mental health practitioner who migrated to England by choice over 20 years ago.

The lived experiences and commitment to human rights and social constructivist standpoints of the researchers may have influenced the interpretation of evidence and the development of concepts. For example, we actively resisted the medicalisation of human experience through our use of strength-based, plain language to frame concepts such as ‘sources of resilience’ and ‘survival strategies’. All decisions about the review process and the interpretation of evidence were made jointly by team members, who contributed their expert opinion and experiential knowledge as equal partners.

### Search strategy

We devised search terms using the PICO approach^38^ (see Supplementary Material available at https://doi.org/10.1192/bjo.2022.51). As the search aimed to be highly sensitive, we employed a search strategy combining search terms for population (e.g. transgender OR ‘gender non-conforming’) and exposure (e.g. migra* OR refugee* OR asylum*). The search terms were adapted for use in nine electronic databases, from database conception to April 2020. These were: MEDLINE, International Bibliography of the Social Sciences, Sociological Abstracts, Web of Science Core Collection, PsycINFO, Embase, CINAHL, Proquest Dissertation and Theses Global. All major journals in the fields of migration and LGBTQ+ health were indexed in the selected databases. Additional search methods included contacting experts in the field, and back and forth reference checking of included studies.

### Study selection

#### Inclusion criteria

Studies were selected if at least 25% of their participants were individuals who identified as transgender and lived as forced migrants in a host country, among the overall sample. In studies with participants of mixed identities, we also required that the data pertaining to the transgender participants could be extracted as primary data.^39^ Where authors gave a definition for transgender, it had to have equivalent meaning to: a person whose gender identity differs from the sex the person had or was identified as having at birth. In our definition of forced migrants, we included refugees, asylum-seekers and individuals who have crossed national borders seeking refuge but do not or cannot seek asylum.

We included any qualitative studies that used both qualitative data collection and analysis methods, as well as autobiographical accounts, published in academic journals or dissertations. Mixed-methods studies that provided qualitative data and analysis specific to transgender participants nested within a broader study were included. Studies published in English and Spanish (the languages spoken by review team members) were included, and salient extracted data were translated into English for synthesis.

#### Exclusion criteria

We excluded studies that collected data using qualitative methods but did not analyse these data using qualitative methods. We also excluded studies that had no primary data, such as literature reviews, descriptive articles, conference abstracts, protocols, and research not about experiences or perceptions.

#### Study selection process

Two authors (S.H. and K.E.) independently screened the titles and abstracts returned by the database search, with more than 95% concordance. They then independently assessed full texts against the inclusion and exclusion criteria. Screening and study selection were reviewed by four authors (B.C., R.B., A.S. and J.S.) at various stages. Disagreements were resolved by seeking additional data or clarification from the authors of included studies on two occasions, and by review team discussion.

### Data extraction

Included papers were read in full by four authors (S.H., K.E., R.B. and B.C.), and data were extracted from studies ordered alphabetically by first author. We extracted data relating to the research team, methods, participants and context. Where multiple publications arose from the same study, the most detailed publication was used for data extraction.

Two authors (S.H. and R.B.) extracted first- and second-order data (themes, subthemes and quotations of author interpretations) into an Excel spreadsheet. Twenty-five per cent of studies were doubly extracted and checked for accuracy; no divergence was found.

### Quality assessment

We used the Joanna Briggs Institute's 2017 Critical Appraisal Checklist for Qualitative Research.^40^ The tool consists of ten criteria (e.g. congruity between the research methodology and the research question, congruity between research methodology and the interpretation of results). Studies were awarded ‘Yes’, ‘No’, ‘Unsure’ or ‘Not applicable’ for each checklist item.

On review of the literature, we felt there were important aspects of quality assessment that were not captured by the JBI checklist. Therefore, we developed and piloted six additional areas to assess. (a) Have issues relating to intersectionality been fully considered? (b) How relevant is the research to the review? (c) Do the authors fairly and comprehensively represent the field using background literature? (d) Have the authors made every effort to ensure fair and equal opportunity of participation? (e) Is there evidence of critical reflection on the role of the study setting and local conditions in shaping the study? (f) Have the participants been appropriately involved in the research? (See Supplementary Material for a breakdown of how each of these areas was developed and assessed.) We did not exclude studies based on our assessment of study quality, as reporting style varied between disciplines and limited reporting does not necessarily equate to unreliable findings. No GRADE-CERqual was undertaken; instead, we accounted for the quality appraisal of studies with our expanded criteria.

All studies were independently assessed by two authors (S.H. and R.B.) with 90% concordance. Discrepancies were resolved by seeking further opinion and consensus from the review team. One review team member (B.C.) authored three of the included publications; this author did not participate in the quality assessment in this study.^41^

### Synthesis approach

#### Process for determining how studies are related

We determined how studies were related by comparing findings that answered the research questions. Two authors (S.H. and R.B.) assigned short codes line-by-line to the extracted first- and second-order data using Excel, enabling the identification of patterns across the data-set. Three authors (S.H., B.C. and J.S.) generated themes and subthemes, over multiple meetings, by carefully reviewing and collapsing codes with shared meanings.^42^ We then related the themes and subthemes back to the review aims, informing the development of overarching categories.

Traditionally, meta-ethnographies determine how studies are related through second-order themes and concepts.^43^ However, distinguishing between first- and second-order interpretations is complex, as narratives are often blurred during the research process (e.g. during the selection of participant quotations and presentation of author interpretation).^43^ The review team prioritised developing and refining themes from first-order participant data; second-order themes were captured separately. Separating the relation and translation of participant data confronts the long history of disempowerment and misrepresentation of the transgender community by prioritising the preservation of participants’ words.^44,45^

#### Process of translating studies

We conducted reciprocal translation across studies using a matrix (Excel), with each study entered into a separate column, and themes and subthemes in separate rows, showing the studies where descriptive themes and subthemes appeared.^46^ Translation used both first-order and second-order themes. A further column captured participant and author quotations that encapsulated the meaning of each theme and subtheme, ensuring that the translation of studies was grounded in the themes developed from the words and statements of the participants in the original publications. Constant referencing back to the original first- and second-order extracted data further grounded the synthesis in the data. The first- and second-order translations were compared to check the credibility of authors’ conclusions. The translation of studies was presented at team meetings to enable critical reflection and discussion of alternative interpretations and explanations.

Where groups of themes refuted the predominant argument presented across the studies, we performed refutational translation. This involved examining the study design, research processes and contextual information (such as the discipline, research aims, and author theoretical and cultural positionality) of studies to explain the inconsistencies in the narratives.

#### Developing the theoretical model

In the final synthesis stage, we aimed to produce a global understanding of the factors impacting mental health outcomes in TFMs. Initially, we synthesised the themes and subthemes into a conceptual map of the review findings, taking into account the relationships between themes that were explored during translation. We then adapted the socio-ecological model^47^ to highlight individual, interpersonal, organisational and societal factors affecting mental health outcomes in TFMs and portray an overarching account of the phenomenon.

## Results

### Outcome of study selection

**Fig. 1:**
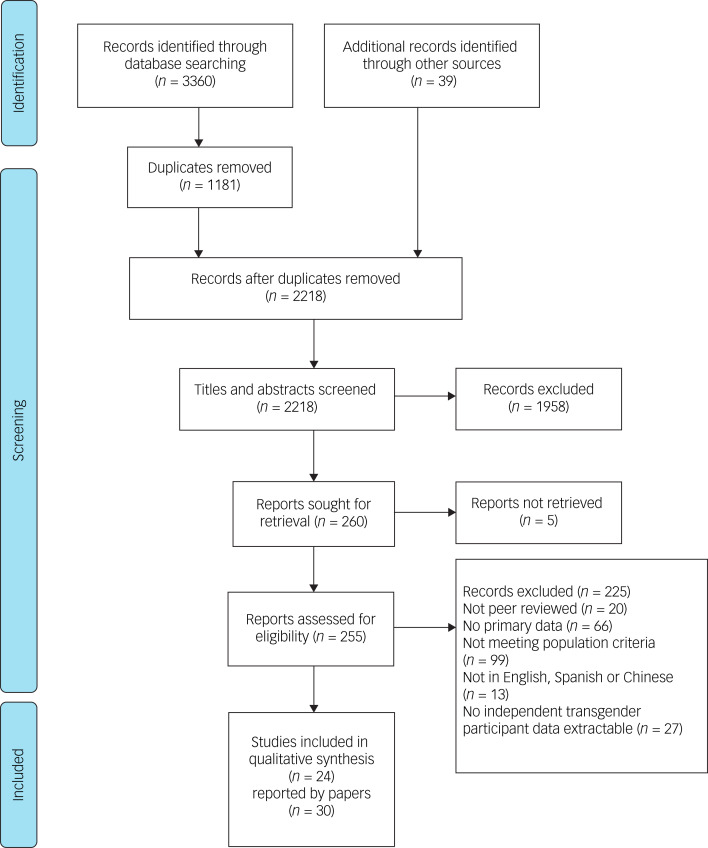
PRISMA flow diagram.

The search retrieved 3399 records initially; 1181 were removed as duplicates. After screening titles and abstracts against our eligibility criteria, we read 260 full-text papers at the final screening stage. Of these, we included 30 papers describing 24 studies. Twenty-two were published in English, and two in Spanish. Six of the papers were doctoral theses; all other included papers were published in peer-reviewed journals. The search process and results are presented in Fig. 1.

### Characteristics of included studies

Table 1 summarises the included studies. Four studies had multiple publications included in the review (studies 2, 5, 21 and 24; Table 1). For study 5, two publications arose from the same study but had no overlap in the research questions asked or primary data used. Therefore, data were extracted from both publications. The studies were from over fifteen disciplines from the health sciences, social sciences and humanities. Research aims were predominantly explorative and focused on the structural, social and cultural determinants of lived experiences of participants from pre to post migration. The theoretical and cultural positionings of the study authors, where reported, showed a broad application of critical theories (including feminist, critical race and intersectionality frameworks) and constructivist perspectives. Most authors who reported their positionalities (authors of 47% of studies) were cisgender, bilingual, immigrants or descendants of immigrants, and experienced in working with marginalised populations. Of those who stated their genders, two authors were transgender (1, 2), one was gender-variant (22) and one was gender non-conforming (18).

**Table 1:** Summary of studies

Study no.	Author(s) and year published	Location of research team	Discipline	No. TFM ppts (total no.)	Gender(s) of participants	Country of origin (no. if reported)	Host country
1	Anonymous (2014)	Uganda	N/A	1	Trans	Rwanda	Uganda
2	Camminga, B (2017; 2017; 2019^a^)	South Africa	Sociology	20	Transgender, Trans man	Central Africa, East Africa, Countries bordering South Africa, Horn of Africa, Southern Africa	South Africa
3	Cashman (2018)	USA	Linguistics	1	Trans woman	Mexico	USA
4	Cerezo, Morales, Quintero and Rothman (2014)	USA	Psychology	10	Transgender women	Mexico (7), Belize (1), Cuba (1), and Honduras (1)	USA
5	Gowin et al. (2017)	USA	Public health	45	Transgender women	Mexico	USA
6	Cisneros and Bracho (2019)	USA.	education	10 (31)	Gender queer (6), transgender women (4)	6 unspecified Latin American countries	USA
7	Engelstein and Rachamimov (2019)	Israel	History	4	Trans women	Israel	France, Belgium, Amsterdam
8	Howe, Zaraysky and Lorentzen (2008)	USA, Mexico	Anthropology	29	Transvestites (3) Women (26)	Mexico	USA, Mexico
9	Hwahng et al. (2019)	USA	Psychiatry	13	Transgender, gender variant	Argentina (1), El Salvador (1), Honduras (1), Mexico (7) and Puerto Rico (1), not specified (2)	USA
10	Martinez (2008)	USA	Social anthropology	4	Transgender (3), transsexual (1)	Mexico	USA
11	Mohyuddin (2001)	USA	Law	2 (4)	Transsexual women	Nicaragua Egypt	USA
12	Mora-Lett (2019)	USA	Social work	20	Transgender women	Honduras (4), Guatemala (3), El Salvador (2) and Mexico (11)	USA
13	Padron (2015)	USA	Chicanx studies	101	Transgender Latinas	Cuba, Dominican Republic, El Salvador, Guatemala, Honduras, Mexico, Peru, Puerto Rico	USA
14	Palazzolo (2016)	USA	Global health	8	Chicas trans	El Salvador (6), Guatemala (1), Puerto Rico (1)	USA
15	Rausenberger (2016)	Belgium	Anthropology	8	Transsexual women (5), male cross-dressers or transvestites (3)	Ecuador (3), Brazil (2), Puerto Rico (1), Peru (1) and Venezuela (1)	Belgium
16	Rhodes et al. (2015)	USA	Health policy	9	Transgender women	Mexico	USA
17	Rosenberg (2016)	Lebanon, Uganda, Ecuador	LGBT health policy	29 (46)	Trans women (25) Transgender	Not reported	Lebanon, Uganda, Ecuador
18	Salas (2019)	Spain	Educational psychology	9	Transsexual (2), woman (2), trans-male (2), non-binary person (1), trans-female (1), male (1)	Mexico (8), Peru (1)	USA
19	Shakhsari (2014)	Netherlands	Political science	4 (10+)	Transgender men (2), transgender women (2)	Iran	Turkey
20	Silva and Ornat (2015)	Belgium	Geography	10	Travestis	Brazil	Spain
21	Van der Pijl et al. (2018) Swetzer (2016)^a^	Belgium	Criminology	6	(Trans)man, transgender women (3), transvestite, (trans)woman	Suriname, Caribbean, Central America, Latin America, Bolivia	Netherlands
22	Vaan Schuylenbergh et al. (2019)	Belgium	Sexual health	49	Female, between male and female, sometimes male, sometimes female and male^b^	Ecuador (28), Colombia (3), Spain (2), Bolivia, Italy, Panama, Romania, Russia, Peru, Venezuela, Netherlands (1)	Belgium
23	Vogel (2009)	Spain, Germany, Venezuela	Anthropology	11	Transformista	Venezuela	Spain, Germany, Italy, Switzerland, France
24	Zarco-Ortiz and Reynosa (2020); Zarco-Ortiz (2018); Zarco-Ortiz (2019)^a^	Mexico	Regional studies	5	Trans women	Guatemala, Honduras, El Salvador	Mexico, US

TFM ppts, transgender forced migrant participants.

a. Primary paper used for data extraction and quality appraisal.

b. Not self-identification as these were prescribed categories in a survey.

The studies included primary data from at least 170 interview participants, 49 asylum declarations, 142 survey participants, nine participants in photo-voice projects and 37 focus group participants. This number is larger when taking into account the more than 50 TFMs who informed ethnographic observations and an unspecified number who took part in focus groups identified as sexual minorities. Study 1 was a personal account written by a TFM and published in an academic journal. The studies took place in locations across North America, Central America, South America, Africa, Europe and the Middle East. Three studies took place in more than one country (8, 15 and 22). Table 1 includes a summary of study design and participant information (see Supplementary Material for further detail).

### Outcome of quality appraisal

JBI quality assessment revealed variation across the 24 included studies (see Supplementary Material for full results). Theses consistently had a high standard of design and reporting quality. As study one was a personal account, it was excluded from the quality appraisal; therefore, the following statistics refer to 23 studies. Thirty-five per cent of studies answered ‘yes’ to five criteria or fewer. Overall, the congruity in the study designs was high, with 75% of criteria about study design answered as ‘yes’ across the studies. The remaining 25% were rated ‘unsure’ owing to insufficient information. Across the studies, the quality of reporting was moderate. Forty-three per cent of studies did not report an analysis method. Sixty-five per cent did not include an ethics statement. Forty-eight per cent of studies did not include a statement locating the researcher culturally or theoretically, and 57% did not address the influence of the researcher on the research and *vice versa*. This can be explained in part by the different expectations and requirements in different academic disciplines.

**Fig. 2:**
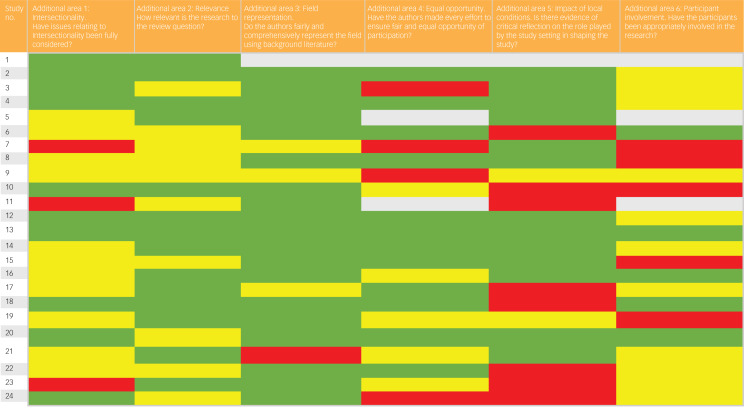
Additional areas of quality appraisal. Green = Yes; Red = No; Yellow = Not sure; Grey = Not applicable.

The additional areas of quality assessment developed by the research team showed varied results across all 24 studies (Fig. 2). In 61% of studies, consideration of intersectionality and use of appropriate and respectful terminology were absent or poorly reported. For the remaining 39% of studies, sample characteristics were fully described using clear and precise language to communicate participant gender self-determination, and authors reflected on intersectionality in the findings and discussion. Forty-two per cent of studies had low overall relevance to the research question, defined as studies that only partially answered the review question. This was often due to only a small portion of included papers reporting relevant primary data. Only 22% of studies had good participant involvement, which we defined as designing and conducting the study to ground the analysis and conclusions in participants’ experiences, and actively involving participants in the design and conduct of the study or data analysis. In 33% of studies, authors did not reflect on the impact of the study setting on their work.

### Overview of main findings

The main findings are organised into 19 subcategories, which are grouped into four categories: stressors, mental health outcomes, sources of resilience and individual survival strategies.

### Stressors

The widespread destitution faced by TFMs results from social and economic marginalisation linked to pervasive and persistent violence, discrimination, unobtainable documentation, barriers to healthcare access and economic exclusion. All subcategories in this section are present for TFMs throughout their lives. Most factors are more influential pre-migration, and the severity of these stressors varies among country of origin and host country.

### Pervasive and persistent violence

Pervasive and persistent violence captures the multiple levels of violence that TFMs experience across their lifespans. We adopted the World Health Organization's definition of violence: ‘the intentional use of physical force or power, threatened or actual, against oneself, another person, or against a group or community, that either results in or has a high likelihood of resulting in injury, death, psychological harm, maldevelopment, or deprivation’.^48^

TFMs reported experiencing long-lasting interpersonal violence from family and intimate partners (studies 1, 2, 4, 5, 10, 12, 13, 14, 16, 18, 19, 20, 23), communities (1, 2, 3, 12, 17, 18, 21), and people in positions of power, including police, teachers, doctors, immigration officers, and traffickers (1, 2, 4, 5, 7, 10, 12, 13, 14, 16, 17, 18, 19, 20, 21, 23, 24). Some TFMs were forced into abusive relationships with boyfriends, landlords, friends and employers (8, 14, 19, 20, 23).

*Being a trans, being a sin! After all these years old, the sun shines every day but never on us. Abused, rejected, harassed and killed. (Transgender person; 1)*

Intense and regular targeted violence, such as physical injury, sexual assault and neglect, from trusted loved ones, community members and people in positions of power contributed to psychological distress and was a driving force behind decisions to migrate.

*In the United States, the gay community is a little more accepted. Like human rights and all that and in my country they are not. There, they will burn you alive because you are trans- gender [sic]. You would be hit, mistreated, your hair cut, they will burn you . . . it's horrible, it's hell on earth. Here, I feel I am free from that threat. (Woman; 4)*

TFMs also described collective social, political and economic violence facilitated by states (1, 2, 3, 4, 5, 6, 9, 10, 11, 12, 13, 14, 15, 16, 17, 18, 19, 20, 21, 22, 23, 24). In countries of origin, state-sanctioned punishments of gender non-conforming behaviour and an absence of legal protections against discrimination and abuse perpetuate a culture that makes life unliveable for many transgender people. Many participants felt dehumanised by this pervasive, persistent and state-engineered violence and discrimination (1, 2, 10, 17, 18, 20). Disempowerment through systemic economic and social exclusion meant individuals felt they had no agency (1, 2, 3, 5, 6, 13, 14, 15, 16, 17, 18, 20, 23, 24). Most participants felt they had no choice but to seek refuge in countries that recognise and protect the existence of transgender people. However, many experienced further traumatisation during their journeys and in their host countries (1, 2, 4, 12, 14, 17, 18, 19, 20, 21, 24).

#### Culture of discrimination

TFMs experience lifelong pervasive and persistent discrimination at school, in healthcare settings, in public places and in the workplace (1, 2, 3, 4, 5, 6, 7, 8, 9, 10, 11, 12, 13, 14, 15, 16, 17, 18, 19, 20, 21, 23, 25). Participants described discrimination by religious and ethnic communities (1, 2, 10, 12, 15, 16, 18) and family rejection of gender non-conforming behaviour (1, 2, 5, 7, 10, 11, 12, 15, 16, 18), which deprived TFMs of socioemotional support from those with whom they shared language and cultural customs:

*There is a [country of origin] community here … and I'm like, ‘Okay, I would like to connect with those people,’ but I'm afraid that they're gonna find out I'm trans … , am [I] going to be physically safe if they find out I am trans? (Trans male; 18)*

The reinforcement of gender norms through discrimination both pre-migration and post-migration generated substantial psychological distress and social isolation, as the discrimination worked to deny individuals’ existence. There was also additional stigma and discrimination around being an ethnic minority post-migration (1, 2, 4, 5, 18, 21), having HIV (9, 10, 13, 14) and facing language barriers (2, 5, 6, 18).

#### ‘The papers make it difficult’

Overwhelming uncertainty is common among TFMs who rely on legal documentation to demonstrate their right to reside in their host country (2, 4, 5, 6, 11, 14, 18). Most narratives came from forced migrants without such documentation. These people depicted lives full of risk-taking to remain in the host country, which was originally thought to be a safe haven, and plagued by uncertainty about their future.

Legal documentation also includes details confirming a person's gender. For transgender people, having identification that indicates a gender incongruous with their gender presentation means they are often rejected from essential services:

*It's like life stops because that is the only thing you use for identification …. this is the thing that everybody, everywhere you go, everybody looks at and looks at you again and questions …  (Trans woman; 2)*

Dependency on congruous gender identity documentation was a major issue for the majority of participants, who had no way of acquiring such documents owing to their forced migration (1, 2, 3, 4, 7, 14, 18, 24).

*If I wanted to change my female status in my ID, I would have to change my birth certificate, so I would have to go back to [my country of origin], which I can't [do]. (Trans male; 18)*

#### Barriers to accessing healthcare

Despite many TFMs citing improved access to healthcare as a key reason for migration, numerous barriers existed in their host countries, including a lack of gender-related care (13, 14, 16, 17, 18, 21, 22) and insufficient or inadequate care provision (1, 5, 12, 14, 16, 17, 18, 19, 21, 22). The need for gender-inclusive care is explained:

*There should be space for us, where we can feel comfortable, where there is a person that is specialized in people like us, where you can go and feel secure and that person knows what you are and isn't going to treat you rudely. (Woman; 16)*

Barriers to accessing healthcare were underscored by the invisibility of transgender people in society, perpetuated by widespread stigma and lack of education about the experiences of transgender people. Another barrier to healthcare access was profound distrust of people in positions of power, including medical professionals, cultivated through individuals’ lived experience of pervasive and persistent violence, leaving TFMs ‘petrified of the healthcare system' (1, 5, 17, 19, 20, 21, 22).

*You don't really want to, it's one of our dreaded fears because then you're ridiculed, and, and… discriminated against. And treated really like you're a disease. (Transgender woman; 21)*

Two authors concluded that formal healthcare could be more difficult to access in host countries, for example, because it was unaffordable or culturally inappropriate (e.g. lack of language support). Consequently, some participants used unofficial pathways to access treatment and care (16, 22), including networks created in transgender communities and the organisations supporting these populations.

#### *‘*Stylists or prostitutes – there are no other options’

Pre- and post-migration economic exclusion refers to the prevention of transgender people from obtaining stable forms of legal income, facilitated by discriminatory hiring practices, low levels of education, an absence of laws protecting equal rights to work, and a dependency on hard to access legal documentation (1, 2, 3, 4, 5, 6, 7, 9, 10, 12, 13, 14, 17, 18, 19, 24). All of these are underpinned by societal stigmatisation of transgender people.

*Employment opportunities are limited. There could be many but there is no education among the citizenry about how to treat a Transwoman [sic]. So, Transwomen [sic] cannot enter the workforce. (Trans woman; 13)*

TFMs were often limited to a small range of low-income jobs including domestic work, the service industry and sex work (2, 4, 5, 7, 8, 9, 12, 13, 14, 15, 17, 18, 19, 20, 21, 22, 23, 24). Many were left unemployed and reported limited access to education and vocational training (1, 2, 4, 5, 6, 9, 12, 13, 18, 23), homelessness and unstable living environments (1, 2, 4, 5, 17, 18, 23) and tremendous suffering including near-starvation (1, 2, 5, 10, 12, 13, 14, 17, 18, 21, 23). *‘I was like a stray dog on the street’ (Woman; 17)*.

In many origin and host countries, sex work was criminalised or culturally disapproved of; however, sex work often remained the most economically viable form of income generation (2, 4, 5, 7, 9, 12, 13, 14, 17, 18, 19, 22, 24). Engaging in sex work meant that transgender women had to face significant health and safety risks. The collective narrative of the women was that they engaged in sex work to survive.

*100 percent of TransLatinas succumb to sex work because there is no other option. (Transgender woman; 13)*

A refuting argument, found in the second-order data of some studies, portrayed sex work as desirable among women and as a factor that drove migration to Europe (8, 15, 20, 21, 23). Reference to the coercive structures, human trafficking, abuse and economic exclusion that push women into sex work was minimal or absent from the authors’ interpretations.

### Mental health outcomes: ‘We are half died’

Embedded in the words of the TFMs was a strong narrative of basic needs going unmet and rights going unfulfilled both in the host country and across the lifespan. The impact of multiple compounding and intersecting stressors was extreme deprivation and suffering, with serious consequences for mental health pre-migration and post-migration.

#### ‘We live in fear’

Living in fear for life is a reality for TFMs who, in their countries of origin, often have no recourse to protection by the state and experience violence at the hands of people in positions of power (1, 2, 3, 4, 5, 10, 12, 13, 14, 15, 16, 17, 18, 19, 20, 23, 24). Living in fear means living with constant hypervigilance, anxiety and psychological trauma, and this endures post-migration. It is then exacerbated by ongoing fears of deportation and a struggle to meet basic needs.

*People can mistreat [and] discriminate against [us], and the law … does not protect [us] … There is no lack of asshole police officer that pick-up transwomen [sic] [that also] abuses [us]. So, life is difficult as a Trans person, and [we] do not know when the ‘lethal game of chance’ will play [us]. (Transsexual woman; 18)*

Avoiding, disengaging, staying away from or dissociating from stressful situations were common survival strategies among TFMs who lived in fear while having no recourse for protection (1, 2, 4, 5, 8, 9, 12, 13, 14, 15, 16, 17, 18, 19, 22, 24). This included substance misuse (5, 9, 13, 16, 18) and long periods of isolation linked to feeling unable to leave the house, form social connections or even apply for asylum (1, 2, 4, 5, 7, 10, 12, 14, 15, 16, 17, 18, 19, 21, 23).

*[In the United States] I did not apply for asylum sooner because I simply could not do it …. I was in a really bad state. I was drinking heavily and doing drugs. I thought it was a way to escape from the trauma and pain of what had happened to me. (Transgender woman; 5)*

*[In Mexico] After the first rape, I was too afraid to leave my house. When I had to leave, I did not go very far. I felt I could not hide who I was so I hid in my house instead. I was afraid the police would find me and rape me again. (Transgender woman; 5)*

#### *‘*I blamed myself for everything’

TFMs also experienced enduring shame and guilt linked to internalised stigma and self-blame for their traumatic experiences and deprivation (2, 4, 5, 7, 10, 11, 12, 14, 16, 18, 21, 23).

*I feel ashamed, I feel guilty for what happened in my life. Rape, maltreatment, and all that … (Woman; 4)*

Self-blame for their circumstances and feelings of worthlessness contributed to exceptionally low self-esteem across the narratives and lifespans (1, 2, 3, 4, 5, 6, 8, 9, 10, 12, 13, 14, 15, 16, 17, 18, 20, 23, 24).

#### ‘I did not see the importance of life’

Feelings of hopelessness, loss of motivation and ‘depression for years and years’ (1, 2, 5, 12, 13, 15, 16, 17, 18) were common among many who faced insurmountable barriers. Some made suicide attempts (1, 5, 10, 12, 18, 19).

*All these things that have happened have given me ideas of suicide so many times. I had grown up being hated by people and finally I came to hate myself! (Transgender person; 1)*

#### *‘*Rejected by everything and everyone’

Isolation due to rejection from family and communities in countries of origin and host countries was common (1, 2, 4, 5, 7, 10, 12, 14, 15, 16, 17, 18, 19, 21, 23). This was exacerbated by a lack of access to stable work, living arrangements and communities, which could have facilitated social interaction and support, and linguistic and cultural differences in host countries.

*I thought no one was ever going to love me. I felt that no one cared about me. (Transgender woman; 5)*

### Sources of resilience

The effects of significant structural and interpersonal stressors were mitigated by family and community acceptance, social belonging, being granted asylum, the fulfilment of basic rights, societal affirmation of gender and healthcare. The sources of resilience held by the individual and offered by communities and host countries provided TFMs with the support and opportunities to survive pre-migration, during migration and post-migration.

#### Family and community acceptance and support

Family and community acceptance and support were powerful mitigators of the stressors faced by TFMs. Although most family members did not accept transgender relatives, those who did typically provided psychosocial support, emotional stability and enhanced self-worth (2, 3, 4, 8, 12, 13, 16, 18, 21, 23). Family support was rare for TFMs before migrating or medically transitioning. However, it seemed to increase following migration, particularly for those who migrated with family or offered economic support to family who remained in countries of origin. LGBTQ+ networks offered spaces of safety, emotional support and gender affirmation (2, 4, 8, 9, 12, 13, 17, 18, 22, 24). Less common, but still sometimes apparent, was local community acceptance at church, at work or within migrant communities in host countries (2, 4, 12, 18).

*I was always going to church and I felt so good. I used to sing in the choir and I just felt that was my protective bubble. (Transgender woman, 12)*

#### *‘*There are people like me’

A sense of social belonging derived from collective identities shared with other transgender people, migrants, ethnic minority groups and trauma survivors helped participants to feel supported in their physical and emotional journeys. Many sought emotional and practical support from members and organisations in their cultural and religious communities (2, 7, 13, 16, 18).

*I think just being able to connect to other queer and trans people and having a community. Being able to support other people that are also going through similar issues like coming out to themselves. (Trans man; 18)*

Most participants identified peer support from other transgender migrants as facilitating resilience against stressors (1, 4, 5, 7, 9, 12, 13, 16, 17, 18, 22, 23). These networks provided financial support, connections to formal legal and social support, unofficial access to healthcare, emotional support, practical advice and a unique sense of belonging born of shared traumas. This support cultivated psychosocial well-being. Meeting in peer support groups appeared to be the most valued form of support experienced by participants:

*It's important that we have these groups because they give us life and they liberate us from our traumas. (Transgender woman; 12)*

#### Societal affirmation of gender

For some people, the attitudes and behaviour of host country organisations and communities provided the acceptance of their gender that many had been deprived of since childhood (2, 3, 4, 7, 8, 9, 12, 13, 15, 16, 18, 21, 22, 24). Gender clinics provided both symbolic and practical gender confirmation, with access reducing psychological distress (2, 3, 5, 7, 8, 9, 11, 12, 14, 16, 18, 19, 20, 21, 22, 23). This external confirmation was partially conferred through legal documentation of gender.

*I was actually looking for freedom and … to enhance my future … the only thing that I knew was that the LGBT community was legalised [in South Africa]. (Transgender woman; 2)*

#### *‘*Te dan el derecho para ser una persona’/‘They give you the right to be a person’

Post-migration fulfilment of basic rights relating to personal safety, income (4, 7, 9, 12, 14, 18) and education (9, 12, 18) brought much-needed relief. Participants spoke of feeling ‘safer’ owing to better legal protections (12, 18, 21, 23).

*Us as trans women, we have learned that we have rights in our jobs and in our homes and that if someone discriminates you or offends you or verbally attacks you, you can now report this so that it can be taken care of. (Trans woman; 12)*

Although formal social support services and educational programmes at times facilitated the fulfilment of basic rights, service use was described infrequently (4, 5, 9, 12, 14, 16, 17, 18), with some fear and distrust of services preventing support-seeking. When people did access services, they went to LGBTQ+ organisations recommended by trusted peers or family members (5).

#### ‘Safe because I've got my asylum’

A minority of TFMs were granted asylum, creating an opportunity for a new life and legalising residency in the host country (2, 10, 14, 18). This led to possibilities for seeking legal employment and better healthcare access. Critically, it removed the fear of deportation to a country they feared they would not survive in.

*That was why I didn't go to school, I wasn't working, and after they approved my asylum case, they sent me a work permit, …. My life changed completely. (Chica trans; 14)*

#### Healthcare access

Although most TFMs continued to struggle to access healthcare in host countries, some found help from a range of services (2, 4, 5, 9, 10, 12, 18, 21), including emergency care, community clinics, psychological therapy, HIV treatment, medication for long-term conditions, gender care and formal support groups.

*[In the United States] I went to a clinic to get hormones … I also got a full body check-up. I got tested for AIDS and other diseases and came out healthy. I had been taking hormones that I bought on the street … (Transgender woman, 5)*

The broader social acceptance of transgender people in host countries was reflected in greater acceptance by healthcare professionals.

### Individual survival strategies

Sources of resilience in communities and host countries were insufficient to overcome the stressors faced, meaning that TFMs had to create strategies to survive. The resilience and resourcefulness of the TFMs in the face of intense adversity was striking. Through perseverance and motivation, many participants worked to build new lives, step by step (4, 7, 9, 12, 15, 18).

*This country gives you many opportunities but having opportunities does not really depend on the country alone. It really depends on you, on how you do it. How have I been able to obtain opportunities? Simply by studying and learning English. [By] working very hard … (Transsexual woman; 18)*

#### *‘*Accept myself as I am’

Self-acceptance and the development of a positive sense of self helped to protect TFMs from the shame and guilt that developed following years of violence and discrimination relating to their gender and their status as forced migrants and ethnic minorities (2, 3, 4, 8, 10, 12, 13, 14, 15, 18, 21, 22, 24).

Self-affirmation of gender and sexuality was an important step towards developing acceptance and self-esteem following traumatic rejection due to gender (2, 3, 4, 8, 9, 12, 13, 14, 15, 21, 22, 23, 24). Although self-affirmation of gender was reinforced by external gender affirmation, the most important element was that TFMs also came to their own affirmation of gender and identity.

*I hear people say [you can't be a lesbian if you are a trans man] such things but for me that doesn't matter because for me my identity is my identity. It's about me, it's about my life. (Trans man; 2)*

#### *‘*Hope and dreams’

Cultivating hope through focusing on the future, focusing on aspects of life where there is a sense of liberation from oppression and nurturing gratitude helped TFMs to remain positive and persevere in dire circumstances (2, 3, 4, 7, 9, 10, 12, 13, 14, 15, 18, 23). Some participants drew strength and hope from their religious beliefs (2, 4, 8).

*I continue to fight the battle and I hope that … well, at least now I see life and enjoy the days and the years. (Transgender woman; 12)*

#### *‘*Purpose in life’

Creating a sense of purpose and reframing themselves as agents in control of their own lives provided psychological relief from a life of powerlessness amid intense oppression (2, 3, 4, 12, 13, 14, 18, 23). Many TFMs felt it was an essential part of their recovery to give back to the communities from which they received life-changing support. The TransLatin@ Coalition in the USA is an example of a transgender migrant-led organisation that empowers TFMs through service provision, advocacy and research (13).

*I want to continue supporting my community. It makes me happy . . . to be helping the girls, even if we can't protect them, we can help empower them to move forward, help them by giving advice. (Woman; 4)*

#### *‘*You cannot let everyone know you are trans’

Ongoing attempts to conceal transgender identity were used as a strategy to keep safe from violence or discrimination (1, 2, 4, 5, 6, 12, 15, 16, 18, 21, 24). Aside from sex work, transgender people often concealed their gender in order to feel safe at work.

*But my biggest fear was … that people would find out and I would get discriminated, that's what it was. I think that was my biggest fear, not being accepted by others and to not have someone to guide and comfort me. (Transsexual woman; 12)*

For many, concealment was essential in their countries of origin, as deep-rooted societal transphobia engendered state sanctioned and interpersonal abuse. Although TFMs believed host countries would offer a safe environment to live openly and access the healthcare necessary to transition, many found that concealment was still necessary in host countries.

### Socio-ecological model of determinants of mental health in TFMs

**Fig. 3:**
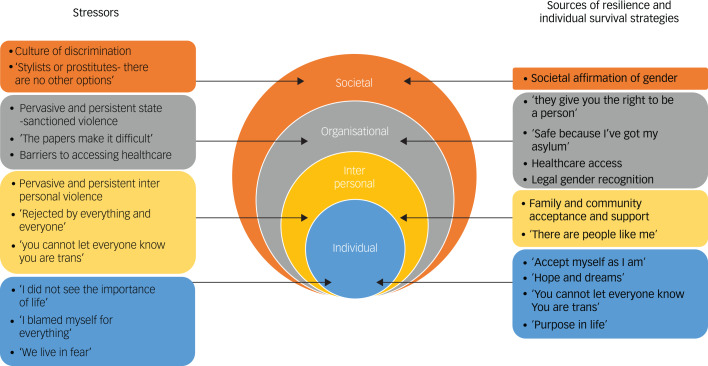
Adapted socio-ecological model of the factors affecting mental health outcomes in transgender forced migrants.

We adapted the socio-ecological model of Bronfenbrenner^47^ (Fig. 3) to distinguish the individual, interpersonal, organisational and societal factors affecting mental health outcomes in TFMs. This model emphasises how the lived experiences of TFMs are embedded within structures and social systems that shape mental health outcomes.

The levels interact with and reinforce one another. For instance, individual survival strategies and sources of resilience from communities may offer TFMs some respite and protection from interpersonal violence and help them to navigate organisational barriers to income and care. Interpersonal stressors are in turn affected by organisational stressors such as an absence of laws protecting transgender people against gender-based discrimination and violence. The socio-ecological model considers the values and beliefs that a society holds, typically communicated through its culture, history and public discourse, which govern the behaviour of the organisations, communities and individuals that exist within it. In a society that values specific gender norms and constructions of the body, and where symbolic language marginalises transgender people, there is a dominant culture that permits discrimination and violence towards members of the marginalised group.

Interactions between the different levels are often shaped by the intersectional identities of individuals. For example, many TFMs, in addition to being a gender minority, are also an ethnic minority in their host country. The intersection of these multiple, oppressed identities may increase the negative impact of interpersonal discrimination on mental health.

## Discussion

### Summary of findings

We synthesised data from 24 qualitative studies exploring the lived experiences of TFMs. The synthesis identified individual survival strategies, as well as factors in interpersonal, organisational and societal environments that contributed to mental health outcomes in this population. The adapted socio-ecological model provided a way to situate and understand the complex, interlinking dynamics of determinants of mental health in TFMs. Our synthesis presents the first detailed global narrative of the lived experiences of TFMs and highlights the need for future research into the ways TFM communities can be protected against stressors and supported in their recovery from trauma.

#### Living with intersecting layers of violence

Our synthesis found that lifelong discrimination and violence are a universal experience for TFMs, underpinned by global societal stigma against transgender people.^49^ Experiences of gender-based violence at the inter-personal and organisational levels are the primary risk factors for poor mental health outcomes in transgender people.^3^ In countries where state-sanctioned violence against transgender people exists, some transgender people are forced to migrate in order to survive. Post-migration, transgender people often do not find sanctuary but instead encounter violence and discrimination resulting from their forced migrant status and their stigmatised gender.^50,51^ Taking an intersectional lens, this synthesis elucidates the compounding impact of having marginalised race, ethnicity and gender on the mental and physical health of forced migrants.^52–55^

#### Community-led intervention

Community-led intervention and peer support from other transgender people, and particularly other forced migrants, appeared to benefit TFMs’ coping capacity, knowledge and access to some vital resources (e.g. hormones). Evidence supports the efficacy of peer support in protecting against poor mental health outcomes for transgender people.^56^ This is particularly important as deeply rooted distrust of people in positions of power, reinforced by systemic discrimination against transgender people in healthcare services, acted as a barrier to accessing inclusive healthcare. Funding for community-led organisations, informed by lived experience, is an essential step in overcoming these barriers.^57^ This has been modelled by the Translatin@ coalition, which combats violence and the incarceration and marginalisation of transgender people across the USA through community programmes.^58,59^ Community-led organisations provide practical and emotional support and opportunities to cultivate individual resilience in the form of hope, self-affirmation, connection and purpose.

#### State-led protection is essential

Individual and community interventions have limitations and were not sufficient on their own to produce positive mental health outcomes. There was no indication from the synthesised literature that individuals’ mental health outcomes improved over time without the fulfilment of basic rights relating to legal status, education, shelter and healthcare. Our findings corroborate evidence that state-level organisational factors relating to legal protection of civil, social, economic and human rights, public policy and service operation, facilitate and sustain health inequities of forced migrants.^11,13,60–62^ For TFMs specifically, dependency on gender-congruent legal documentation was necessary to accessing stable employment, housing and other services. Economic exclusion tied to these organisational factors leaves TFMs little choice but to turn to sex work, which intensifies risks to mental and physical health, particularly in countries where it is criminalised.^63^ This strongly suggests the need for intervention at multiple levels, including legal protection for healthcare access, protection for educational and occupational rights regardless of immigration status, and easy access to immigration support and legal documentation.

#### Prioritising collaborative, lived experience research

Although the findings offer a consistent, global picture of the lived experiences of TFMs, the quality appraisal, particularly around the additional areas we identified, revealed limitations of the current evidence base. Equal opportunity of participation of TFMs in included studies was poorly considered. Overall, researchers reported limited meaningful engagement with the communities they researched. Very often, recruitment of participants relied on chance encounters and access being granted by gatekeepers. We found that in the majority of cases, research was done to people rather than with them, by research teams who did not share an identity or any experiences with participants. This means we cannot know that the findings accurately reflect the priorities and experiences of TFMs, because the research questions and interpretations of data may have been developed from an unacknowledged cisgender, Western standpoint.^64^ Collectively, there was limited, if any, effort to produce influential work beyond an academic exercise to make sustainable changes to the TFM population.

### Strengths, limitations and reflexivity

The use of multidisciplinary literature was both a strength and a limitation of this review. As a strength, synthesising data from a broad range of disciplines diversified the perspectives and priorities of the primary researchers and thus the types of research questions asked. Included studies focused on particular health issues pertinent to the TFM population, explored key concepts relevant to particular phases of life (such as gender concealment and intersecting marginalised identities) and documented life narratives. The multidisciplinary literature enabled us to build a socio-ecological model of the factors affecting mental health outcomes in TFMs. Including studies in Spanish further expanded the reach of the primary data and credibility of the global picture produced. Conversely, different disciplines have different reporting conventions, which can complicate judgements and comparisons of quality. The heterogeneity of studies also prevented the in-depth evaluation of the effects of contextual factors such as dominant country of origin or host country culture and asylum policies on the lived experiences of individuals. We did not complete searches in databases that specifically index journals in languages other than English, as we did not have the resources available. This means that caution should be taken when generalising the findings of this review to communities and countries not represented in the reviewed studies.

Multidisciplinarity was further enhanced by our team, which included researchers and clinicians with varying biomedical, sociological, psychological, experiential and methodological expertise. Data synthesis incorporated perspectives from lived experience researchers and experts in systematic reviews and qualitative methods, enabling us to develop a critical group consciousness towards research prioritising the lived experiences of transgender people, particularly those who have experienced severe trauma and forced migration. This influenced us to honour the self-determination and emphasise the original words of the participants in our reporting. Our open discussions about the discrimination and disempowerment experienced by transgender communities, transgender research participants (at the hands of researchers) and transgender researchers themselves enabled us to grow as individuals and to broaden our understanding of what it means to carry out inclusive and respectful research in a contested space.

### Recommendations and conclusions

This synthesis responds to calls to emphasise the roles of organisational and societal contexts in the mental health outcomes of forced migrants.^50,62,65^ The findings suggest that in host countries, at the individual and interpersonal levels, there is a need for interventions designed to create change in knowledge, beliefs and social relationships. Our synthesis suggests that resilience-based models of intervention that further support the resourcefulness, determination and hopefulness of TFMs might be effective. These could include the use of peer support groups to promote strength through community building, to help TFMs find relief from the emotional scarring caused by lifelong abuse, and to support them in moving toward self-acceptance and finding freedom from shame and guilt. Research into the evolving mental health support needs at specific points in the lifespan of TFMs is needed to ensure that interventions are relevant and timely.

More importantly, at the organisational level, public policy informed by interdisciplinary research that promotes sensitive integration of forced migrants into host country communities, the provision of avenues for legal gender recognition in host countries, wider access to healthcare and trauma-informed service models are needed. Further research into how existing mental health services can meet the needs of this population is required. At the societal level, leaders in the public and private sectors should work with transgender and migrant community leaders to create dialogue and collaboration, to help facilitate understanding and bolster community social support. Improved and nuanced communication and knowledge about the unique needs and concerns of TFMs through intervention at the individual, interpersonal, organisational and societal levels are necessary to improve mental health outcomes.

## Data Availability

The data that support the findings of this study are available from the corresponding author (S.H.) upon reasonable request.

## Supplementary material

For supplementary material accompanying this paper visit http://doi.org/10.1192/bjo.2022.51. click here to view supplementary material
