# Supplementary material for: Lived experiences of transgender forced migrants and their mental health outcomes: systematic review and meta-ethnography
Source: BJPsych Open. 2022 May 10;8(3):e91. doi: 10.1192/bjo.2022.51 (PMC9169499; doi:10.1192/bjo.2022.51)
Supplement: Supplementary file 1 [file S2056472422000515sup001.docx]

**Supplementary information**

| Table 1 | | | |
| --- | --- | --- | --- |
| Search strategy | | | |
| Population | Intervention/Exposure | Comparison | Outcome |
| transgender OR “gender non-conforming” OR “gender nonconforming” OR “gender dysphoria” OR transsexual OR transvest* Or male-to-female OR female-to-male OR transwom?n OR transman OR transmen OR “trans wom?n” OR “trans m?n” OR transfemale OR “trans female” OR transmale OR “trans male” OR transfeminine OR transmasculine OR non-binary OR agender OR bigender OR genderfluid OR “gender fluid” OR genderqueer OR “gender queer” OR intersex OR “inter-sex” OR “sex reassignment” OR “gender change” OR “gender minority” OR “gender diverse” OR travesti OR travestis OR travestismo OR transgénero OR two-spirit OR “two spirit” OR “cross dresser” | migra* OR immigra* OR refugee* OR asylum* OR displaced OR refugiado* OR asilo OR desplazado | N/A | N/A |

| Table 2  Summary of studies | | | | | | | |
| --- | --- | --- | --- | --- | --- | --- | --- |
| Study no. | Study author(s) and year published | Country | Discipline | Research aims | No. TFM participants (Total participant no.) | Data collection method | Data analysis method |
| 1 | Anonymous (2014) | Uganda | N/A | Not applicable | 1 | Personal account | N/A |
| 2 | Camminga, B (2017; 2017; 2019*) | South Africa | Sociology | ﻿Explores terms, such as ‘transgender’, travel, how they are taken up, and to what effect, particularly in relation to the materiality of both the bodies and the circumstances of people in local contexts. | 20 | Life story interviews | No analytic process described |
| 3 | Cashman (2018) | U.S.A. | Linguistics | Examines the concepts of the body, time, space, and transition brought along into, or about, in the narratives | 1 | Observation, interviews | Sociocultural linguistic approach to narrative analysis |
| 4 | Cerezo, Morales, Quintero, and Rothman (2014) | U.S.A. | Psychology | Explores what motivated women to migrate to the United States as well as their negotiation of multiple culturally marked identities in key institutions. | 10 | Semi-structured interviews | Case study approach |
| 5 | Gowin et al. (2017) | U.S.A. | Public Health | Explores preimmigration experiences of violence and postimmigration health status in male-to-female transgender individuals from Mexico applying for asylum in the United States.  Provides insight into the various stressors experienced by transgender Mexican immigrants who seek asylum in the United States, highlights the health consequences of the stressors they experienced in Mexico and in the United States, provides guidance to public health practitioners on ways to address gaps in services. | 45 | Provision of asylum declarations | Grounded theory |
| 6 | Cisneros and Bracho (2019) | U.S.A. | Education | Explores the ways that undocuqueer immigrants negotiate the boundaries of social performance by revealing or concealing their gender, sexuality, and immigration status. | 10 (31) | Semi-structured interviews | Unspecified, identified themes |
| 7 | Engelstein and Rachamimov (2019) | Israel | History | Charts the early history of the Israeli transgender community from the first public campaign in 1953 to the mid-1980s. | 4 | Oral history interviews | No analytic process described |
| 8 | Howe, Zaraysky, and Lorentzen (2008) | Mexico and U.S.A. | Anthropology | Illustrates some of the structural, social, and cultural factors that condition male-to-female transgender sex workers’ lives. | 29 | Interviews | No analytic process described |
| 9 | Hwahng et al. (2019) | U.S.A. | Psychiatry | ﻿Examines socio-cultural determinants of health such as standards of living, institutions, political/economic structures, health behaviours and psychosocial factors related to substance use, mental health, and HIV risk, prevention and management. Explored how participation in harm reduction groups helped mitigate the stressors trans Latina immigrants encountered. | 13 | Focus groups | Thematic analysis |
| 10 | Martinez (2008) | U.S.A. | Social Anthropology | ﻿Explores the universes of sense and meaning through the stories of four Mexican women, three transgender and one transsexual, infected with HIVAIDS in Mexico and who are currently seeking political asylum in San Diego, California. | 4 | Life story interviews and observation | Phenomenology and semiotics |
| 11 | Mohyuddin (2001) | U.S.A. | Law | Discusses the development of United States asylum law' and its recognition of sexual minorities. | 2 (4) | Provision of asylum declarations | No analytic process described |
| 12 | Mora-Lett (2019) | U.S.A. | Social Work | Explores the various systems and intersections that impact gender identity and the migration experiences of Trans Latina Immigrants. Also to explore the factors that influenced TLI’s decision to immigrate to the U.S. and their pre-migration, during-migration, and post- migration experiences. | 20 | Semi-structured interviews | Grounded theory |
| 13 | Padron (2015) | Belgium | Chicanx Studies | Explores why TransLatinas migrate, what there perspective is on their migration to the U.S. and their current social conditions in this country. Explores how they navigate and resist systemic oppression and explore how to foster spaces that prolong and sustain rather than shorten and suspend TransLatina lives and life stories. | 101 | Survey | No analytic process described |
| 14 | Palazzolo (2016) | U.S.A. | Global Health | Explores the contextual factors that determine or mitigate vulnerability to HIV among Latina transgender women. | 8 | In-depth interviews | Unspecified, identified themes |
| 15 | Rausenberger (2016) | Ecuador, Lebanon, Uganda | Anthropology | Explores who the Latin American transgender sex workers in Antwerp are, why they work in the sex industry, which journey brought them to Europe, how they experience life, how they feel about their bodies, identities and sexuality, and how art is used as a creative coping strategy to escape their remarkable reality. | 8 | Semi-structured interviews, informal conversations,observation | No analytic process described |
| 16 | Rhodes et al. (2015) | U.S.A. | Health Policy | Documents the needs, assets, and priorities of immigrant Latina transgender women using photovoice. | 9 | Photo-discussions, community forums | Grounded theory |
| 17 | Rosenberg (2016) | Turkey | LGBT Health Policy | Discusses the particular issues faced by trans* refugees and summarise suggestions for responding to those issues. | 29 (46) | Focus groups and in-depth interviews | No analytic process described |
| 18 | Salas (2019) | Spain | Educational Psychology | Explores the experiences that Trans Latinx immigrants have as a result of holding membership in multiple oppressed social groups. To understand how Trans Latinx immigrants cope with their experiences belonging to multiple oppressed social groups. | 9 | Semi-structured interviews | CQR analysis method |
| 19 | Shakhsari (2014) | Netherlands | Political Science | ﻿Explores the conditions of queer and trans refugee lives in Turkey or the ‘‘third country of asylum’’ are and how these conditions engender a tempo of rights where certain acts marked as the ‘‘violation’’ of human rights and when and where are they seen as individual crimes. | 4 (10+) | Ethnography interviews, observation | No analytic process described |
| 20 | Silva and Ornat (2015) | Belgium | Geography | ﻿Highlight the ways in which Brazilian travestis negotiate and strategise transnational mobility in order to work as prostitutes in Spain. | 10 | In-depth interviews, observed 50 people | Content analysis |
| 21 | Van der Pijl et al. (2018) Swetzer (2016)* | Belgium | Criminology | ﻿Explores how Latin American and Caribbean transwomen compared to Dutch transwomen experience the healthcare system in the Netherlands. | 6 | Semi-structured interviews | Cross-case synthesis |
| 22 | Vaan Schuylenbergh et al. (2019) | Spain, Germany, Venezuela | Sexual Health | Explores the prevalence of uncontrolled gender-affirming hormone use, silicone injections, and inconsistent condom use among transgender sex workers working in Antwerp, Belgium. | 49 | Structured survey interviews; In-depth interviews | Descriptive analysis of survey; grounded theory |
| 23 | Vogel (2009) |  | Anthropology | Explores how and why transformistas arrived at their present situation as sex workers in Europe. | 11 | Biographical interviews, observed 40 people | No analytic process described |
| 24 | Zarco-Ortiz and Reynosa (2020); Zarco-Ortiz (2018); Zarco-Ortiz (2019)* | Mexico | Regional Studies | Analyses the migration process of transgender women from Central America to Tapachula, Chiapas. To understand the transformative processes of the body linked to displacement and the manifestations of gender. To analyse the ways in which these trans women modify and produce territoriality, and finally, distinguish the security and sexual relation strategies with the processes of trans-border migration control. | 5 | Observation and semi-structured interviews | Análisis NIP |

| Table 3  Participant information | | | | | | | | |
| --- | --- | --- | --- | --- | --- | --- | --- | --- |
| Study no. | Gender of participants | Age ranges | Ethnicity | Country of origin | Host country | No. years in host country | Socio-economic status indicator | Legal status in host country  (no., if reported) |
| 1 | Trans | 32 | Not reported | Rwanda | Uganda | Not reported | Homelessness and low educational attainment | Not reported |
| 2 | Transgender, Trans man | 20-50 | Not reported | Central Africa, East Africa, Countries bordering South Africa, Horn of Africa, Southern Africa | South Africa | 5+ | Not specified (majority unemployed or had low income jobs) | Not specified (some undocumented  some came with visas) |
| 3 | Trans woman | 50 | Latinx | Mexico | USA | 20 approx. | Not specified, low income jobs | Unauthorised migrant status |
| 4 | Transgender women | 26-54 | Latinx | Mexico (7), Belize (1), Cuba (1), and Honduras (1) | USA | 5-31 (range), 11 (mean) | 50% employed (domestic work (2), service industry (1), and outreach in the non-profit sector (2))  50% unemployed  90% lived below national poverty line ($10,890 annual income) | Not specified (some had legal status, asylum granted,  mismatched documentation) |
| 5 | Transgender women | 20-58 | Not reported | Mexico | USA | Not reported | (65%) some or all high school  (13%) education beyond high school (11%) unknown | Not specified (all applied for asylum) |
| 6 | Gender queer (6), transgender Women (4) | 19-30 | Latina | Unspecified 6 Latin American countries | USA | Not reported | Not specified | Legal residential status (2), undocumented (29) |
| 7 | Trans women | Not reported | Not reported | Israel | France, Belgium, Amsterdam | Not reported | Not specified | Not reported |
| 8 | Transvestites (3)  Women (26) | Not reported | Not reported | Mexico | USA and Mexico | Not reported | Low socioeconomic backgrounds, 100% engaged in sex work. Majority finished high school. | 100% Undocumented |
| 9 | Transgender, Gender variant | ﻿22-50 | Latina (12) White (1) | ﻿Argentina (1), El Salvador (1), Honduras (1), Mexico (7) Puerto Rico (1), unspecified (2) | USA | Not reported | 38% did not graduate high school 50% full-time legal employment in previous year | Not specified, majority undocumented |
| 10 | Transgender (3)  Transsexual (1) | 20-45 | Not reported | Mexico | USA | Not reported | 100% had university degrees | Undocumented |
| 11 | Transsexual women | Not reported | Not reported | Nicaragua Egypt | USA | 10+ | Not specified | Granted asylum |
| 12 | Transgender women | ﻿24-53 | Not reported | ﻿Honduras (4), Guatemala (3), El Salvador (2) and Mexico (11) | USA | ﻿1-10 (range), 8 (mean) | Not specified, majority had low income jobs (house cleaning, restaurants, hair salons, nightclub, sex work,peer educator)  20% unemployed | ﻿Legal status (3), political asylum (11), Visa (1), other immigration benefit (1), undocumented (1) or declined to answer (3) |
| 13 | Transgender Latinas | 29-50 | Latina | Cuba, Dominican Republic, El Salvador, Guatemala, Honduras, Mexico, Peru, Puerto Rico | USA | Not reported | 51% were employed.  91% did not have jobs which provided medical insurance. | Not reported |
| 14 | Chicas trans | 25.9 (mean) | Not reported | El Salvador (6), Guatemala (1), Puerto Rico (1) | USA | 9.5 (mean) | 50% did not complete high school 100% were employed | Granted asylum (2), applied for asylum (2), undocumented (3), citizenship (1) |
| 15 | ﻿Transsexual women (5), Male cross-dressers, Transvestites (3) | 39-52 | Not reported | Ecuador (3), Brazil (2), Puerto Rico (1), Peru (1) and Venezuela (1) | Belgium | Not reported | Not specified, 100% engaged in sex worker | Not reported |
| 16 | Transgender women | ﻿22-45 | Latina | Mexico | USA | 4-16 (range), 10 (mean) | 44% did not finish high school ﻿55% reported at least high school diploma or passing the general educational development test.  66% full-time employment (working in restaurants, cutting hair, and cleaning houses)  33% had part-time employment (factory, cashier, gardener). | Not reported |
| 17 | Trans women (25)  Transgender | Not reported | Not reported | Not reported | Lebanon, Uganda, Ecuador | Not reported | ﻿Not specified, majority of women engaged in sex work. | Not reported |
| 18 | Transsexual (2) Woman (2) Trans-male (2) Non-binary person (1) Trans-female (1) Male (1) | ﻿23-48 | Latinx (6) Latina (1) Mexican (1) Hispanic (1) | Mexico (8), Peru (1) | USA | ﻿ 6 -30 (range), 15.2 (mean) | ﻿ 11% did not complete high school. | Undocumented (5), documented (2), granted asylum (1), DACA (1) |
| 19 | Transgender men (2) Transgender women (2) | Not reported | Not reported | Iran | Turkey | Not reported | Not specified but implied poverty and engagement in sex work. | Asylum seekers |
| 20 | Travestis | ﻿20-38 | White (1) Not reported | Brazil | Spain | Not reported | Not specified, low levels of formal education, low incomes.  80% engaged in sex work. | Undocumented |
| 21 | (trans)man, Transgender women (3),  Transvestite, (trans)woman | ﻿26-42 | Not reported | Suriname, Caribbean, Central America, Latin America, Bolivia | Netherlands | 20 (one participant) | 50 % unemployed.  17% engaged in sex work. | Asylum seekers (3), unreported (2), citizenship (1) |
| 22 | Female, between male and female, sometimes male, sometimes female and male* | Not possible to disentangle trans participant ages | Not reported | Ecuador (28), Colombia (3), Spain (2), Bolivia, Italy, Panama, Romania, Russia, Peru, Venezuela, Netherlands (1) | Belgium | Not reported | 50% did not complete high school | Citizenship (3), Spanish citizenship (20) |
| 23 | Transformista | Not reported | Not reported | Venezuela | Spain, Germany, Italy, Switzerland, France | 10+ (one participant) | Not specified, 100% engaged in sex work | Legal status (1), otherwise undocumented or not reported |
| 24 | Trans women | 21-34 | Not reported | Guatemala, Honduras, El Salvador | Mexico, US | 0.3- 12 (range) | Not specified, majority engaged in sex work | Undocumented (not specified), legal status through family (1), refugee (1) |
|  |  |  |  |  |  |  |  |  |

*not self-identification as these were prescribed categories on a survey

## JBI Critical Appraisal Checklist for Qualitative Research

| **Table 2**  JBI Critical Appraisal Checklist for Qualitative Research | | | | | | | | | | | | |
| --- | --- | --- | --- | --- | --- | --- | --- | --- | --- | --- | --- | --- |
| Study no. | Item 1 | Item 2 | Item 3 | Item 4 | Item 5 | Item 6 | | Item 7 | | Item 8 | Item 9 | Item 10 |
| 1 |  |  |  |  |  |  |  | |  | |  |  |
| 2 |  |  |  |  |  |  |  | |  | |  |  |
| 3 |  |  |  |  |  |  |  | |  | |  |  |
| 4 |  |  |  |  |  |  |  | |  | |  |  |
| 5 |  |  |  |  |  |  |  | |  | |  |  |
| 6 |  |  |  |  |  |  |  | |  | |  |  |
| 7 |  |  |  |  |  |  |  | |  | |  |  |
| 8 |  |  |  |  |  |  |  | |  | |  |  |
| 9 |  |  |  |  |  |  |  | |  | |  |  |
| 10 |  |  |  |  |  |  |  | |  | |  |  |
| 11 |  |  |  |  |  |  |  | |  | |  |  |
| 12 |  |  |  |  |  |  |  | |  | |  |  |
| 13 |  |  |  |  |  |  |  | |  | |  |  |
| 14 |  |  |  |  |  |  |  | |  | |  |  |
| 15 |  |  |  |  |  |  |  | |  | |  |  |
| 16 |  |  |  |  |  |  |  | |  | |  |  |
| 17 |  |  |  |  |  |  |  | |  | |  |  |
| 18 |  |  |  |  |  |  |  | |  | |  |  |
| 19 |  |  |  |  |  |  |  | |  | |  |  |
| 20 |  |  |  |  |  |  |  | |  | |  |  |
| 21 |  |  |  |  |  |  |  | |  | |  |  |
| 22 |  |  |  |  |  |  |  | |  | |  |  |
| 23 |  |  |  |  |  |  |  | |  | |  |  |
| 24 |  |  |  |  |  |  |  | |  | |  |  |

Green = Yes, Red = No, Yellow = Unsure, Grey = Not applicable

### Checklist items

1. Is there congruity between the stated philosophical perspective and the research methodology?

2. Is there congruity between the research methodology and the research question or objectives?

3. Is there congruity between the research methodology and the methods used to collect data?

4. Is there congruity between the research methodology and the representation and analysis of data?

5. Is there congruity between the research methodology and the interpretation of results?

6. Is there a statement locating the researcher culturally or theoretically?

7. Is the influence of the researcher on the research, and vice- versa, addressed?

8. Are participants, and their voices, adequately represented?

9. Is the research ethical according to current criteria and is there evidence of ethical approval by an appropriate body?

10. Do the conclusions drawn in the research report flow from the analysis, or interpretation, of the data?

## Development of additional areas for quality appraisal

All additional areas were assessed with a “yes”, “no”, “unclear”, “not applicable” rating. For areas with multiple sub-criteria (1,4,6) the majority of criteria had to be fulfilled to be awarded “yes”. If 50% or less of the sub-criteria were fulfilled, studies were awarded “unsure”.

1. Have issues relating to intersectionality been fully considered? (a,b,c, adapted from Sweeney et al., 2019)
   1. Have sample characteristics been fully described, including age and ethnicity, gender?
   2. Has diversity of experience been explored in the findings in relation to intersectionalities?
   3. Have issues relating to intersectionalities been explored in the discussion or limitation sections?
   4. Have the authors used clear and precise language to communicate the self-identification of their participants?
2. How relevant is the research to the review? (Brunton, 2011)
   1. Does this study help to answer the review question?
3. Do the authors fairly and comprehensively represent the field using background literature?
4. Have the authors made every effort to ensure fair and equal opportunity of participation?
   1. Have the researchers made reasonable effort to reach the potential participants and to inform them of the study?
   2. Do the researchers factor language and other accessibility requirements into their study and procedure?
   3. Do the researchers attempt to accommodate participant needs e.g. meeting them at a time and place that they are comfortable with?
5. Is there evidence of critical reflection on the role played by the study setting and local conditions in shaping the study?
6. Have the participants been appropriately involved in the research? (adapted from Harden et al., 2006; Sweeney et al., 2019; Thomas et al, 2003)
   1. Does the study use appropriate data collection methods to ensure findings are grounded in participant's experiences?
   2. Are the methods appropriate to ensure that data analysis is grounded in participant's views?
   3. Are participants actively involved in the design and conduct of the study?
   4. Are participants involved in data analysis (e.g. member checking, reviewing transcript)

References

Sweeney A, Clement S, Gribble K, Jackson E, Carr S, Catty J & Gillard S (2019) A systematic review of qualitative studies of adults’ experiences of being assessed for psychological therapies. *Health Expectations*22(2): 133-148. Received a certificate of achievement for being a top downloaded publication.

Thomas J, Sutcliffe K, Harden A, et al. *Children and Healthy Eating: A Systematic Review of Barriers and Facilitators.* London, UK: EPPI-Centre, Social Science Research Unit, Institute of Education, University of London; 2003.

Harden A, Brunton G, Fletcher A, Oakley A, Burchett H, Backhans M. *Young People, Pregnancy and Social Exclusion: A Systematic Synthesis of Research Evidence to Identify Effective, Appropriate and Promising Approaches for Prevention and Support.* London, UK: EPPI-Centre, Social Science Research Unit, Institute of Education, University of London; 2006.

Brunton G, Wiggins M, Oakley A. *Becoming a Mother: A Research Synthesis of Women's Views on the Experience of First-time Motherhood.* London, UK: EPPI Centre, Social Science Research Unit, Institute of Education, University of London; 2011.
